# Telomere length regulates ISG15 expression in human cells

**DOI:** 10.18632/aging.100066

**Published:** 2009-07-17

**Authors:** Zhenjun Lou, Jun Wei, Harold Riethman, Joseph A. Baur, Regina Voglauer, Jerry W. Shay, Woodring E. Wright

**Affiliations:** ^1^ Department of Cell Biology, The University of Texas Southwestern Medical Center at Dallas, Dallas, TX 75390, USA; ^2^ Molecular and Cellular Oncogenesis Program, The Wistar Institute, Philadelphia, PA 19104, USA; ^3^ Current address: Department of Physiology, University of Pennsylvania School of Medicine, Philadelphia, PA 19104, USA; ^4^ Department of Biotechnology, Institute of Applied Microbiology, University of Natural Resources and Applied Life Sciences, A-1190 Vienna, Austria

**Keywords:** telomere position effect, ISG15, aging, cancer, cell turnover, inflammation

## Abstract

Endogenous genes regulated by telomere length have not previously been identified in human cells. Here we show that telomere length regulates the expression of interferon stimulated gene 15 (ISG15, 1p36.33). ISG15 expression (RNA and protein) increases in human cells with short telomeres, and decreases following the elongation of telomeres by human telomerase reverse transcriptase (hTERT). The short-telomere-dependent up-regulation of ISG15 is not mediated by replicative senescence/DNA damage signaling or type I interferons. In human skin specimens obtained from various aged individuals, ISG15 is up-regulated in a subset of cells in older individuals. Our results demonstrate that endogenous human genes can be regulated by the length of telomeres prior to the onset of DNA damage signals, and suggest the possibility that cell turnover/telomere shortening may provide a mechanism for adjusting cellular physiology. The upregulation of ISG15 with telomere shortening may contribute to chronic inflammatory states associated with human aging.

## Introduction

The ends of human chromosomes (telomeres) consist of many kilobases of the  repeating DNA sequence TTAGGG, ending in ~18 to 600 nucleotides of single-stranded G-rich repeats [1-4]. This 3' overhang is proposed to  be inserted into the double-stranded DNA, forming a local D-loop and an overall structure called a t-loop [5,6]. In combination with the binding of telomeric proteins, this structure is thought to hide the ends of the chromosomes from being recognized as double-strand breaks needing repair by NHEJ, which would form dicentric chromosomes and cause a mitotic catastrophe [7,8].

A combination of incomplete replication, processing events and oxidative damage shortens telomeres with each round of cell division. This shortening is prevented in the germline and certain stem cells [9] by the presence of telomerase. Telomerase is a ribonucleo-protein. The RNA component hTR (hTERC) [10] contains a C-rich sequence that serves as a template for the addition of TTAGGG repeats using the reverse transcriptase activity contained within the protein catalytic subunit hTERT [11,12]. Telomerase activity is repressed in most human somatic tissues during development [13], leading to progressive telomere shortening with  subsequent cell divisions. When telomeres become short enough to produce inadequate telomeric protein binding or t-loop packaging they generate a DNA damage signal, which causes the growth arrest known as senescence or replicative aging [8]. Many other stimuli can also induce an irreversible growth arrest which has historically been called senescence even though the arrest is not telomere based [14].

In addition to protection of linear chromosome ends, telomeres may also be involved in the regulation of gene expression. The evidence comes from experiments in which a reporter gene inserted next to a natural or an artificial telomere results in  repression of expression of the reporter gene, a phenomenon called telomere position effect (TPE). Telomere position effect (TPE), first described in *Drosophila*, can result in the silencing of genes positioned next to telomeres [15,16]. TPE has been described in a variety of organisms, including *Saccharomyces cerevisiae* [17], *Saccharomyces pombe* [18], *Trypanosoma brucei* [19,20], *Plasmodium falciparum* [21], mice [22] and humans [23,24]. In *S. cerevisiae*, there appears to be two different mechanisms of TPE [25]. "Classical" TPE is dependent on the *SIR* family of proteins, and usually spreads in a continuous fashion for several kb into the subtelomeric region. A second mechanism involving HAST domains (Hda1-affected subtelomeric) influences the expression of genes ~10-25 kb from the telomeres. There is evidence suggesting that both of these mechanisms may respond to nutrient deprivation or stress, in which relief of TPE contributes to the upregulation of a variety of subtelomeric genes (reviewed in [25]).

How telomere length might regulate gene expression in mammals is completely unknown. The efficiency of TPE on model reporters placed next to healed chromosomes in human cells varies with telomere length [24]. In contrast to yeast and parasites, where telomere length is thought to be relatively constant in normal cells, telomere length decreases with age in humans, raising the intriguing possibility that telomeric regulation of gene expression might have a different   function in mammals. Replicative senescence has been shown to be associated with DNA damage signals from "too-short" telomeres [26,27], so there is no reason to suspect that TPE is involved in senescence. However, there is currently no demonstrated mechanism by which cells monitor the length of their telomeres prior to their becoming short enough to generate a DNA damage signal. We have speculated that telomere length changes in TPE might be a mechanism for using cell turnover to monitoring long periods of time (years or decades) in order to coordinate life-history strategies in long-lived organisms [28]. Similarly, length-regulated TPE might be used to change gene expression in tissues undergoing areas of chronically increased cell turnover due to inflammatory or other processes, to adjust the physiological response over time. Either of these hypotheses predicts that the number of genes regulated by telomere length might be small, since it would not represent a general mechanism of gene regulation used during development and normal physiology but only in special circumstances.

In previous studies, reporter genes and artificially truncated telomeres were used to demonstrate that telomere length could play a role in the repression of reporter gene expression in mammals [22-24]. No endogenous genes next to telomeres have yet been shown to be regulated by telomere length in human cells. None of 34 telomere-proximal genes were found to vary with telomere length when young and senescent human fibroblasts were compared [29]. Telomere-proximal genes have been poorly represented in microarry chips because the complicated repeat nature of the subtelomeric region delayed completion of the human genome sequence to the very ends of the chromosomes until recently. In order to perform a more comprehensive search for genes regulated by telomere length, we constructed a microarray chip containing many newly identified telomere-proximal genes. We examined gene expression patterns in a variety of cell types in which we had manipulated telomerase in order to dissociate telomere length changes from other confounding factors such as time in culture and DNA damage signals from short telomeres. We here report the identification of *ISG15* (Interferon Stimulated Gene 15kda) as the first endogenous human gene whose expression is regulated by telomere length. *ISG15* is a stress-response gene that may function as a tumor suppressor and contributor to inflammatory responses [30]. This raises many intriguing issues concerning the role of telomere length prior to replicative arrest in the physiology of human aging.

## Results

### Identification of genes up-regulated with telomere shortening

Table 1 lists a panel of human fibroblasts and mammary epithelial cells with variations in telomere lengths used in the present studies. To examine the correlation of gene expression and telomere shortening, we used a "Telo-Chip", a customized microarray containing 1,323 potential subtelomeric genes (within 1,000 kilobase pairs from the telomeres) representing all 92 telomere ends. The Telo-Chip also contained 92 random control genes, 12 housekeeping genes and 198 other genes (GEO Datasets, GSE6799). The initial screen was performed using total RNA extracted from proliferating young cells with long telomeres (BJ-18, HME31-26, and IMR90-26), cells proliferating with short telomeres (BJ-78, HME31E6/7-69, IMR90-62, and IMR90E6/7-84), and cells proliferating with experimentally elongated telomeres (BJ18hTERT-148, HME31hTERT-68, and IMR90hTERT-134) (Table 1). Probes were hybridized to the Telo-Chips, and data were analyzed with GeneSpring software. Approximately 24 genes that showed at least a 1.5-fold increase in all the cells with short telomeres were further examined using quantitative PCR (q-PCR).

**Figure 1.**
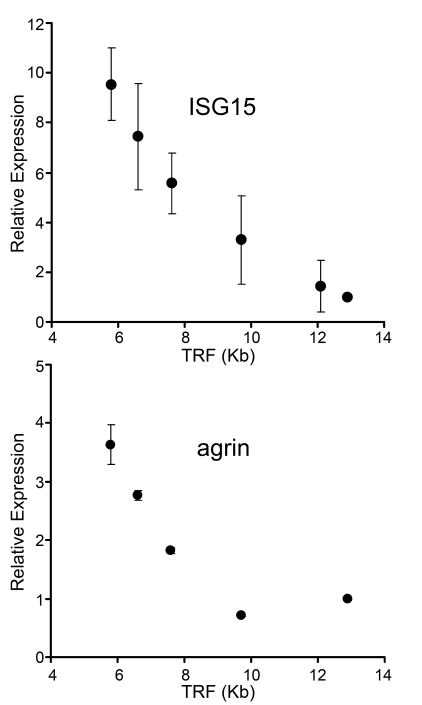
Up-regulation of gene expression with telomere shortening in human fibroblasts. mRNA levels of ISG15 and agrin were assayed by  qantitative PCR in human fibroblasts with different telomere lengths. Results from at least three separate experiments are shown as means ± SEM. GAPDH was used as an internal normalization control. All values were then normalized to the level (=100%) of mRNA in young cells with long telomeres (PD18) (see Table 1). Results show increase of ISG15 and agrin expression with telomere shortening. Similar results for ISG15 were also obtained in IMR90 cells, NHK and HME epithelial cells (Supplementary Figure 1).

A major problem with previous efforts to identify TPE regulated genes is a failure to distinguish telomere length effects from multiple confounding influences, such as the length of time in culture, DNA damage signaling from short telomeres and clonal succession [31] in heterogeneous primary cultures. Only a single gene,* interfereon stimulated gene 15 (ISG15)*, met our criteria for being regulated by telomere length when examined using the series of BJ fibroblasts with different natural and manipulated telomere lengths (see below). The up-regulation of ISG15 mRNA was also observed in a human lung fibroblast cell line (IMR90) and in human breast and kidney epithelial cell lines (HME31 and NHK) with short telomeres (see Supplementary Figure 1). ISG15 is a ubiquitin-like molecule that can be conjugated to other proteins (reviewed in [30,32]) to modify their function [33]. Figure 1 compares the behavior of *ISG15* with *Agrin*, one of many genes that failed to meet our criteria. Agrin is a neuronal aggregating factor [34] and was selected for illustrative  purposes because it is located just centromere-proximal to *ISG15* on chromosome 1p36.33. The mRNA levels of both ISG15 and agrin increase in human skin fibroblasts as their telomeres shortened with population doublings between PD18 and PD83 in culture (Figure 1).

### Up-regulation of agrin expression is associated with DNA damage signaling and/or replicative senescence 

**Figure 2.**
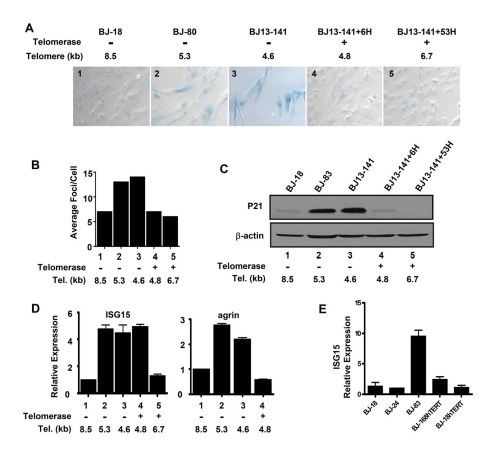
Replicative senescence and DNA damage signaling independent up-regulation of ISG15 expression in cells with short telomeres. (**A**) Short and long-term expression of hTERT rescued cells from replicative senescence. BJ cells with short telomeres (BJ-80 and BJ13-141) exhibited significant increases in the number of SA-β-Gal positive cells; whereas, the cells with long telomeres (BJ-18) did not show SA-β-Gal staining. Exogenous telomerase rapidly eliminated senescent cells (BJ13-141+6H, when only the shortest telomeres had been lengthened) as well as after bulk telomere elongation had occurred (BJ13-141+53H). Rare fields with an SA-β-Gal staining positive cell were selected for the last two images to validate the staining procedure. The number in each image is a key to the cell lines used in B-D. (**B**) γ-H2AX staining shows that exogenous hTERT rapidly eliminates DNA damage signalling due to short telomeres. Approximately 500 nuclei of each cell line were analyzed using Metasystems software (Metasystems, Germany). (**C**) Western blot shows that p21, a transcriptional target of DNA damage-induced p53 signaling, rapidly disappeared following the introduction of telomerase to elongate the shortest telomeres. (**D**) Q-PCR showing that ISG15 expression remained high in BJ cells rescued from replicative senescence/DNA damage signaling after only a few doublings in the presence of exogenous telomerase when telomeres were still short (ISG15, column 4), while elongation of the telomeres after 53 doublings led to decreased expression (ISG15, column 5). In contrast, elimi-nating replicative senescence/DNA damage following a short exposure to telomerase caused a decrease in the expression of agrin (agrin, column 4). Agrin thus did not meet our criteria for telomere length regulation, since its increase in old cells (agrin, columns 2&3) is secondary to senescence and/or DNA damage (column 4). (**E**) BJ cells overexpressing hTERT and having long telomeres express low levels of ISG15.

As cells age, telomeres shorten and the cells eventually enter replicative senescence due to p53-mediated DNA damage signaling from the shortest telomeres [27]. This raised the possibility that damage signaling contributed to the up-regulation of gene expression we observed in cells with short telomeres. In order to examine this question, we elongated the shortest telomeres by expressing hTERT in human fibroblasts BJ13-141. BJ13 is a clone in which telomerase flanked by loxP sites was expressed for seven doublings (beginning at population doubling (PD) 85, approximately five doublings before the parental culture senesced). After seven doubling hTERT was excised by cre-recombinase. The preferential elongation of the shortest telomeres conferred an extra 50 doublings before this clone senesced at PD145 with a telomere length of ~4 kb rather than the usual ~6 kb length at senescence of normal BJ fibroblasts [35]. We characterized the cells both shortly after re-introducing hTERT (early expression, before significant telomere elongation had occurred) and again ~50 doublings later (long-term expression, after telomeres were significantly elongated). The expression of the senescence marker SA-β galactosidase (Figure 2A) and γH2AX DNA damage foci (Figure 2B) increased as cells approached senescence in both BJ-80 and BJ13-141 cells. Expressing hTERT rapidly eliminated the SA-β-gal staining and reduced the γH2AX foci to the levels of "young" cells (Figure 2A and B), even after only six doublings (BJ13-141+6H) when the average telomere length was still close to that of the cells at PD141 (Table 1), indicatingthe expression ofhTERT eliminated the DNA damage signaling and replicative senescence induced by short telomeres. Similarly, the expression of the p53 transcriptional target p21 was elevated in the two near senescent cell types, and its expression also disappeared in cells with both transient (BJ13-141+6H) and long-term (BJ13-141+53H) introduction of hTERT (Figure 2C), further confirming the inhibition of DNA damage signaling from short telomeres by the expression of hTERT.

**Table 1.** Cell lines and strains with different telomere length and telomerase activity. # Cell lines and strains used for Microarray analysis. * TRAP = telomeric repeat amplification protocol. + telomerase positive and – telomerase negative. † Telomere length was determined by Southern blot analysis (TRF). kb, kilobase, NA, not available. § PD = population doublings.

**Human Cell Lines and Strains**	**Description**	TRAP*	**Telomere Length (kb)^†^**
**Skin Fibroblasts**			
			
BJ-18^#^	PD 18^§^	-	12.9
BJ-24	PD 24	-	12.1
BJ-50	PD 50	-	9.7
BJ-72	PD 72	-	7.6
BJ-83^#^	PD 83	-	5.8
BJE6/7-78	Expressing oncoproteins E6/E7 to block p53/pRB signaling, PD 78	-	NA
			
BJhTERT-168^#^	Expressing hTERT, PD 168	+	13.4
BJ18hTERT-148	Expressing hTERT, PD 148	+	10.6
BJ13-141	Near senesence with very short telomeres, PD 141	-	4.6
BJ13-141+6H	6 PD after introducing hTERT into BJ13-141 cells to remove DNA damage signaling and replicative senescence, still with short telomeres	+	4.8
BJ13-141+53H	53 PD after introducing hTERT into BJ13-141 cells, with elongated telomeres, PD 194	+	6.7
BJB14-411	Minimal expression of hTERT, short telomeres, PD 411	+	2.7
BJB14-411+5H	5 PD after introducing hTERT into BJB14-411 cells to remove DNA damage signaling and replicative senescence, still with short telomeres	+	2.7
BJB14-411+50H	50 PD after introducing hTERT into BJB14-411 cells, with elongated telomeres	+	4.8
**Lung Fibroblasts**			
IMR90-24.6^#^	PD 24.6	-	9.9
IMR90-61.7^#^	PD 61.7	-	6.8
IMR90E6/7-84.1^#^	Expressing oncoproteins E6/E7 to block p53/pRB signaling, PD84.1	-	7.2
IMR90hTERT-124.6	Expressing hTERT, PD 124.6	+	13.3
**Mammary Epithelial Cells**			
HME31-25.9^#^	PD25.9	-	3.9
HME31E/6-69.3^#^	Expressing oncoproteins E6/E7 to block p53/pRB signaling, PD69.3	-	2.6
HME31hTERT-67.7^#^	Expressing hTERT, PD67.7	+	3.7

**Figure 3.**
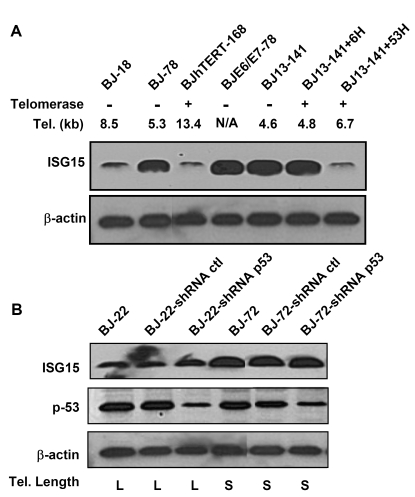
p53 is not involved in the up-regulation of ISG15 expression in cells with short telomeres. (**A**) Western blot of ISG15 in human fibroblasts with different telomere lengths. Both free and conjugated (data not shown) ISG15 increase with telomere shortening (lanes 1 and 2) and in cells with short telomere (BJ13-141 before and after expressing telomerase for 6 doublings, lanes 5 and 6). Expression of HPV16 E6, which degrades p53, had no effect on ISG15 protein expression, while elongation of telomeres by the expression of telomerase for 53 doublings returned ISG15 levels to baseline. β-Actin served as a loading control. A typical result from three independent experiments is s shown. (**B**) Western bolt analysis of ISG15 and total p53 protein in young and old human fibroblasts with long and short telomeres, respectively. Stable expression of shRNA led to significant (> 80%) reduction in the level of p53 protein compared to those in parental and mock infection cells in both young and old cells. The reduction of p53 protein levels had no effect on the expression of ISG15. β-actin served as a loading control.

We then examined the expression of ISG15 and agrin in BJ13 cells early and late after expressing of hTERT. The mRNA level of agrin was up-regulated in cells with short telomeres (Figures 1B and 2D). However, the expression of agrin decreased in BJ13-141+6H cells (Figure 2D), suggesting the up-regulation of agrin in cells approaching senescence was due to DNA damage signaling perhaps as part of  replicative senescence. This pattern was typical of most of the 22 other genes that did not fit our criteria for co-regulation by telomere length. In contrast to agrin, in spite of the elimination of DNA damage signaling and replicative senescence, ISG15 expression (mRNA in Figure 2, protein in Figure 3) remained at a high level in BJ13-141+6H cells, indicating that the up-regulation of ISG15 expression in cells with short telomeres was not due to DNA damage signaling and/or replicative senescence.

### Telomere length is involved in the regulation of ISG15 expression

To examine the role of telomere length in the regulation of ISG15 expression, we cultured the BJ13-hTERT cells for an extended period to elongate the telomere length. After 53 population doublings (BJ13-hTERT+53H), the average telomere length was elongated from 4.6kb (BJ13-141) to 6.7kb (Table 1). With telomere elongation, the ISG15 expression decreased to the level of young cells (Figure 2D, lane 5 and Figure 3A), sug-gesting that ISG15 expression is associated with telomere length in human fibroblasts. This is further confirmed by the observation that populations of BJ fibroblasts overexpressing hTERT that had long telomeres (Table 1) expressed ISG15 at levels of young cells (Figure 2E and 3A).

**Figure 4.**
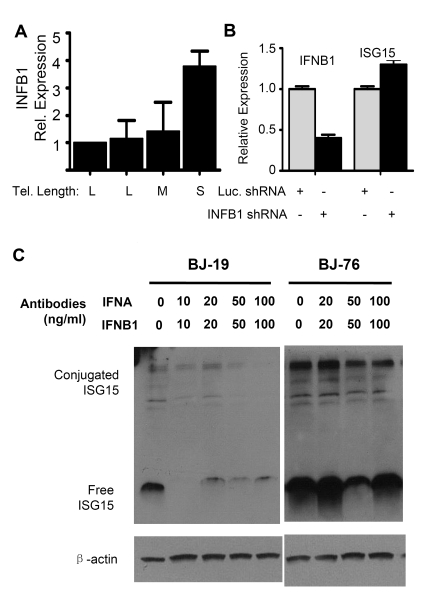
The up-regulation of ISG1 *5* expression in cells with short telomeres does not depend on interferon beta1 (INFB1). (**A**) Q-PCR analysis of mRNA levels of INFB1 in human fibroblasts with different telomere length (BJ-18 to BJ-83). Results from at least three separate experiments are shown as means ± SEM. GAPDH was used as an internal normalization control. All values were then normalized to the level (=100%) of mRNA in young cells (PD18) with long telomeres. Results show an increase of INFB1 expression with telomere shortening. (**B**) Stable knock down of INFB1 by shRNA in BJ cells with short telomeres did not reduce the expression of ISG15. mRNA levels of ISG15 and INFB1 were quantified by q-PCR. (**C**) Western blot showing that blocking antibodies to both IFN α and β reduced the levels of both free and conjugated ISG15 in young BJ fibroblasts, but failed to reduce expression in old cells with short telomeres. Young (BJ-18) and old (BJ-76) cells were treated with neutralizing antibodies against INFA and INFB1.

### Up-regulation of ISG15 with telomere shortening is independent of the expression of p53 and type I interferons

ISG15 expression can be regulated by genotoxic stress [36-38]and type I interferons [32]. The up-regulation of ISG15 in the BJ fibroblast series is not associated with p53 expression and function as shown by knocking-down the expression of p53 in both young and old BJ cells. Reducing p53 levels had no effect on ISG15 protein expression  (Figure 3B).  Similarly, blocking  of p53 functions by overexpression of E6 had no effect of ISG15 expression (Figure 3A, lane 4).

Q-PCR of *interferon α *(INFA) showed no change in expression between young and old cells (data not shown) while *interferon β1* (INFB1) increased (Figure 4A). Stably reducing INFB1 expression by ~60% in PD76 BJ cells using shRNA produced no change in the expression of ISG15 (Figure 4B), indicating that the increase in ISG15 expression in cells with short telomeres is not a result of increased interferon expression. This is further confirmed by the fact that adding blocking antibodies to both INFA and INFB1 to the medium failed to reduce ISG15 expression in old cells. However, the antibodies did reduce ISG15 expression in young (PD19) BJ fibroblasts (Figure 4C), suggesting that much of the basal level of ISG15 expression in young cells is secondary to the interferons they secrete. However, the increase seen in cells with short telomeres is controlled by an independent mechanism other than up-regulation of type I interferon expression, since blocking antibodies failed to affect expression.

### Up-regulation of ISG15 with aging in human skin biopsies

**Figure 5.**
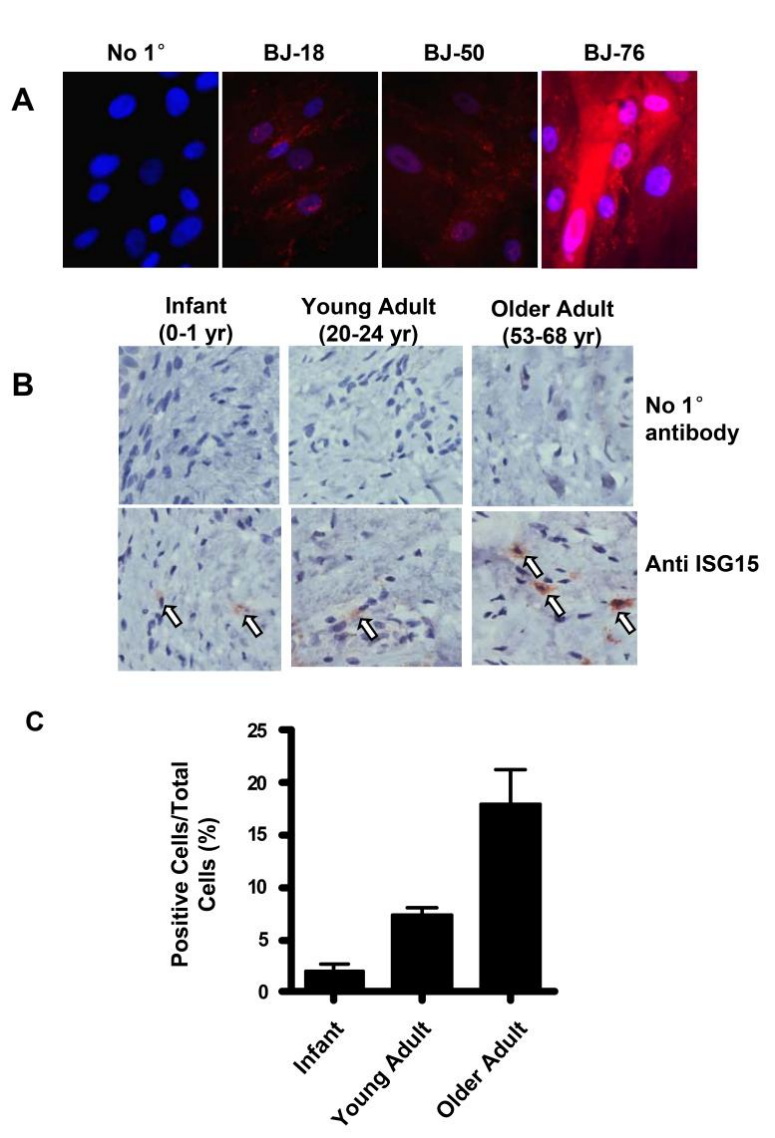
ISG15 is increased in human skin with aging. (**A**) Immunofluorescence staining of ISG15 in BJ cells at different population doublings. The negative sample was treated identically except no primary antibody was added. Nuclei were stained with DAPI. Staining intensity increases in cells with short telomeres. (**B**) Immunochemical staining illustrating an age-dependent up-regulation of ISG15 expression in the dermis of human skin tissues. 2-4 cases were examined in each group. Infant, 0-1 year old; young adult, 20-24 year old; older adult, 53-68 year old. No primary antibody was added to the negative control. (C) Quantitation of the results of all the samples described above. 8-10 random fields were counted for each sample.

Significant up-regulation in ISG15 protein expression with telomere shortening was also confirmed by immunofluorescence staining (Figure 5A) in human skin fibroblasts. Intense staining was observed in the cells with short telomeres at PD78. Since telomeresshor- ten with *in vivo *aging [39], we also examined ISG15 expression in human skin tissues from donors of different ages (Figure 5B). The older adult group (ages 53-68) showed a significant increase in the number of ISG15-positive cells in the dermis (Figure 5C), indicating that up-regulation of ISG15 can occur *in vivo*.

### Expression of ISG15 is not regulated by "classical" TPE

*ISG15* is located on 1p36.33, about 1M base pairs from the telomere. In yeast, classical TPE spreads in a continuous fashion from the telomere, so that all of the genes between a TPE silenced gene and the telomere would also be silenced. We examined eight other genes located between *ISG15* and the telomere, and none of them showed a correlation between gene expression and telomere length in BJ cells (Supplementary Figure 2). Whether *ISG15* is regulated by telomeric looping or indirectly by other telomere-length regulated genes remains to be determined.

## Discussion

In this study, we analyzed more than 1,300 subtelomeric genes and open reading frames (ORFs) and another 300 genes of interest using a customized microarray chip. Using young (long telomeres) and old (short telomeres) human cells we found genes whose up-regulation is associated with DNA damage and senescence when telomeres shorten, e.g. *Agrin*. We also demonstrated that up-regulation of ISG15 with telomere shortening was not due to genotoxic stress but correlated with telomere length, providing evidence that in addition to protecting linear chromosome ends, telomeres are also involved in the regulation of gene expression in human cells.

ISG15 [40] is the founding member of a family of ubiquitin-like modifiers (Ubls) that include SUMO, NEDD8, HUB1, APG12 and APG8 [41,42]. ISG15 has the structure of a di-ubiquitin molecule, and the E1 and E2 enzymes that prepare ISG15 for conjugation [UBE1L [43] and UBCH8/UBE2L6 [44]] are also ubiquitin-conjugating enzymes. Although there appears to be common pathways involving ubiquitin and ISG15, conjugation does not increase the degradation of ISGylated proteins [45,46]. Although many targets of ISGylation have now been identified [45], the consequences of modification by ISG15 are in most cases unknown. ISG15 is able to inhibit viral release by blocking the neddylation of viral assembly proteins [47]. In addition, free ISG15 can be secreted [48] and there is evidence that it has cytokine-like immuno-modulatory properties [49-51].

There are three properties of ISG15 that make it particularly intriguing as a gene regulated by telomere length. Most of the genes that are regulated in yeast by TPE can be characterized as being related to stress/nutritional deprivation [25]. ISG15 is expressed as part of the innate immunity/stress response pathway, and its regulation by telomere length is consistent with a conservation of telomeric input into the control of some aspects of the stress response. Secondly, secreted ISG15 may contribute to age-related inflammation. ISG15 can stimulate the production of the proinflammatory interferon, IFNγ, in CD3+ cells [49,50]. Although IFNγ alone did not stimulate the proliferation and cytotoxicity of NK cells in mixed cultures with CD3+ lymphocytes, their growth and non-MHC-restricted killing activity was greatly increased following exposure to free ISG15 [49]. Unconjugated ISG15 is also a chemotactic factor for neutrophils [51]. Collectively, these results suggest that secreted ISG15 could contribute to a pro-inflammatory environment. It is well known that  inflammation is associated with a large number of age-related physiological conditions, such as sarcopenia [52], atherosclerosis and cardiovascular diseases [53,54]. In addition, neurodegenerative diseases [55,56], renal failure [57], osteoporosis [58], and the metabolic syndrome/diabetes [59] are associated with inflammation, and it has been suggested that dysregulation of inflammation is a fundamental aging mechanism [60-62].

 The role of replicative aging in human aging has long been debated, and the relatively low number of senescent cells in tissues from elderly donors has been used as an argument that it is not relevant to organismal aging. However, it is well established that telomere length declines with age in a large number of different human tissues [39]. Since ISG15 expression increases progressively with decreasing telomere length before cells become senescent (Figure 1A), the effects of replicative aging/telomere shortening on organ function could also be exhibited before cells became senescent.

Finally, there is suggestive evidence that ISG15 can function as a tumor suppressor [30]. Premalignant cells have to undergo many divisions before they become invasive, and it is thought that the primary function of replicative aging is to limit the number of available divisions as a brake against cancer formation [63]. Furthermore, hyperproliferation provides one of the conditions favoring the development of malignancies. It is possible that the increased ISG15 expression that accompanies proliferation induced telomere shortening functions to create an internal or external environment that restricts tumor progression. Terminal telomere shortening has been viewed as a "two-edged sword", since in the absence of p53 very short telomeres can cause genomic instability and may contribute to the formation of cancer. ISG15 regulation may be one mechanism by which telomere shortening suppresses tumor formation prior to the telomeres becoming sufficiently short to cause problems of genomic instability.

At least five genes between *ISG15* and the telomere were not regulated by telomere length (Supplementary Figure 2). Discontinuous TPE can occur in yeast [64,65], and DNA looping [66] might be one mechanism that TPE could extend 1Mb from the telomere to the *ISG15* gene  without controlling the expression of the intervening genes. Alternatively, telomere length could control *ISG15* indirectly by affecting a different telomeric gene that we have not yet identified. Regardless of whether it is direct TPE or not, the consistent change in gene expression that we observe when we manipulate telomeres establishes that *ISG15* is regulated by telomere length.

The fact that only a single gene, *ISG15*, was identified in this study is not unexpected. There are reasons to suspect cell-type specific variation in the expression of telomere-length co-regulated genes [67]. In the absence of gene regulation by telomere length cells would lack the ability to monitor telomere length prior to the point when they become so short that they generate a DNA damage signal. The role of telomere shortening in monitoring cell turnover or the passage of time may vary in different tissues. There may be many other genes that are tissue-specific and only expressed in particular cell types as a function of telomere length.

The finding that ISG15 expression correlates to the telomere length in human cells suggests that telomeres are involved in the regulation of gene expression and may be involved in broader physiological functions beyond the protection of the linear ends of chromosomes. Shortening of telomeres occurs with aging both *in vivo* and in cell culture. It will be of great interest to explore the role of ISG15 in tumor suppression and age-associated inflammatory condi-tions, and finally to identify additional genes and pathways regulated by telomere length and how they impact human biology.

## Methods

Microarray analysis. Microarray analysis was carried out at the UT Southwestern Medical Center at Dallas DNA Microarray Core Facility (http://microarray.swmed.edu/). Briefly, 60-70 nt oligo-nucleotides representing the selected genes were synthesized (Operon Biotechnologies, Inc.) and printed on glass slides (Cat. PXP-U 50B, Full Moon BioSystems, CA). Total RNAs isolated from human skin fibroblast (BJ-19, BJ-78, BJ18hTERT-148 and BJhTERT-168), human lung fibroblasts (IMR90-26.4, IMR90-61.7, IMR90E6/7-84.1 and IMR90hTERT-134.6) and human mammary epithelial cells (HME31-25.9, HME31E6/7-69.3 and HME31hTERT-67.7) were used to make fluorescence-labeled cRNAs for hybridization by following the procedure provided by the Microarray Core Facility (http://microarray.swmed.edu/protocols/General_spotted_array.htm). The slides were scanned and the data were analyzed using GeneSpring software.

Quantativite PCR. Quantatitive PCR was carried out using the human Universal Probe Library (Cat. 04683633001) and TaqMan Master (Cat. 04535286001) from Roche following the manufacture's manual. The experiments were repeated 2-3 times, and the relative expression level of each gene was normalized to the young cells with long telomeres (BJ-18, IMR90-24.6, and HME31-25.9). The primers and probe for each gene were selected by using ProbeFinder software provided by Roche (https://www.roche-applied-science.com/sis/rtpcr/upl/adc.jsp). GAPDH was used as loading control.

Western blot analysis. Western blot analysis was carried out as described [68]. Monoclonal antibody against human ISG15 was generously provided by Dr. Ernest Borden (Cleveland Clinic Foundation, 1:1,000). Antibody against p53 was purchased from Calbiochem (OP-43, 1:1,000), and antibody against ß-actin was from Sigma (A1978, 1:20,000).

shRNAs. The retroviral-vector based shRNA construct against human p53 gene was provided by Dr. J.D. Minna (Hamon Center for Therapeutic Oncology Research and Departments of Internal Medicine, Pharmacology,UT Southwestern Medical Center at Dallas). The retroviral-vector based shRNA constructs against human INFβ1 gene were purchased from OpenBiosystems (Cat. RHS1764-97198161, RHS1764-97197488, and RHS1764-9206903).

## Supplementary figures

Supplementary Figure 1 ISG15 expression in other cells lines. The expression of ISG15 in other cell lines with long (young and hTERT expression) and short (old) telomeres. PCR reagents was analyzed by q-PCR using probes from Roche Applied Science. The relative levels are normalized to that in young cells for each cell type. Telomere length was determined by Southern blot analysis (TRF). (**A**) IMR 90 lung fibroblasts. (**B**) HME31 mammary epithelial cells. (**C**) NHK human kidney epithelial cells.

Supplementary Figure 2 Expression of other 1p subtelomeric genes in BJ cells with different telomere lengths. q-PCR was used to examine eight genes between ISG15 and the telomere. No signals were detected for genes XM_001127463, XM_926974 and XR_015286, so only five genes are shown to compare with ISG15. PCR reagents and probes were from Roche Applied Science. GAPDH was used as an internal normalization control. The relative levels are normalized to that in young BJ cells. The numbers in parentheses indicate the distance of the gene from the 1p telomere (**A**) ISG15, (**B**) NM_018948, (**C**) XR_015292, (**D**) XR_017611, (**E**) NM_001005484, (**F**) XR_017612.
