# Supplementary figures and images for: Telomere length regulates ISG15 expression in human cells
Source: Aging (Albany NY). 2009 Jul 17;1(7):608–21. doi: 10.18632/aging.100066 (PMC2806043; doi:10.18632/aging.100066)

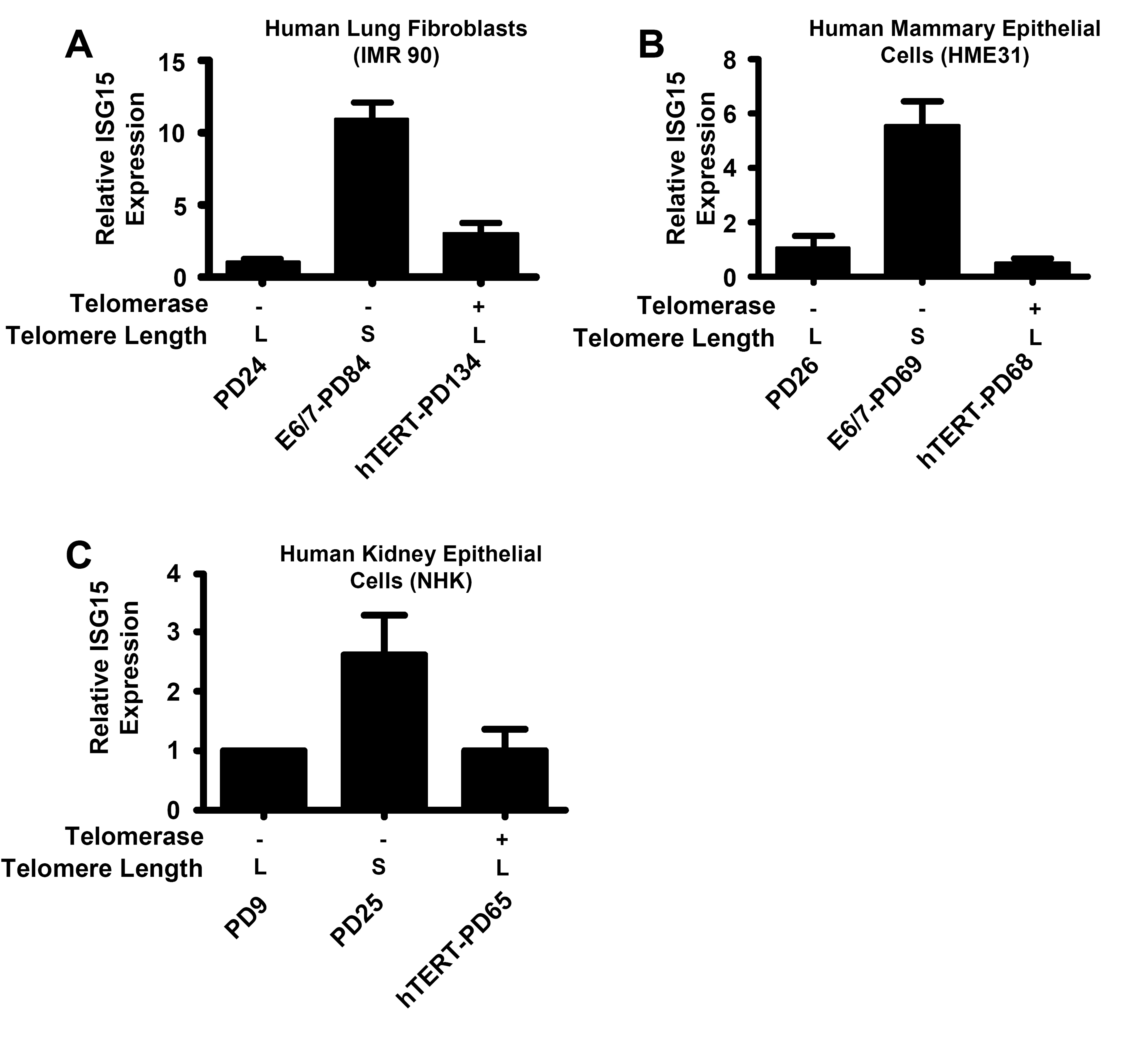

Supplement: Supplementary Figure 1 — The expression of ISG15 in other cell lines with long (young and hTERT expression) and short (old) telomeres. PCR reagents was analyzed by q-PCR using probes from Roche Applied Science. The relative levels are normalized to that in young cells for each cell type. Telomere length was determined by Southern blot analysis (TRF). (A) IMR 90 lung fibroblasts. (B) HME31 mammary epithelial cells. (C) NHK human kidney epithelial cells. [file aging-01-608-s001.tif]
